# Intensity of Glycemic Exposure in Early Adulthood and Target Organ Damage in Middle Age: The CARDIA Study

**DOI:** 10.3389/fphys.2021.614532

**Published:** 2021-06-23

**Authors:** Yifen Lin, Xiangbin Zhong, Zhenyu Xiong, Shaozhao Zhang, Menghui Liu, Yongqiang Fan, Yiquan Huang, Xiuting Sun, Huimin Zhou, Xingfeng Xu, Yue Guo, Yuqi Li, Daya Yang, Xiaomin Ye, Xiaodong Zhuang, Xinxue Liao

**Affiliations:** ^1^Department of Cardiology, First Affiliated Hospital of Sun Yat-sen University, Guangzhou, China; ^2^NHC Key Laboratory of Assisted Circulation, Sun Yat-sen University, Guangzhou, China

**Keywords:** hyperglycemia, left ventricular hypertrophy, cardiac dysfunction, coronary artery calcium, albuminuria

## Abstract

**Aim:**

To determine whether long-term intensity of glycemic exposure (IGE) during young adulthood is associated with multiple target organs function at midlife independent of single fasting glucose (FG) measurement.

**Methods:**

We included 2,859 participants, aged 18–30 years at Y0, in the Coronary Artery Risk Development in Young Adults (CARDIA) Study. IGE was calculated as the sum of (average FG of two consecutive examinations × years between the examinations) over 25 years. Target organs function was indicated by cardiac structure, left ventricular (LV) systolic function, LV diastolic function, coronary artery calcium (CAC), and urine albumin-to-creatinine ratio (UACR) at Y25. We evaluated the associations between IGE with target organs function using linear regression models and estimated the associations between IGE with numbers of organs involved (0, 1, or ≥ 2 organs) using multinomial logistic regression models.

**Results:**

A 1-SD increment of IGE was significantly associated with worse target organs function after multivariable adjustment: left ventricular mass (β [SE], 5.468 [1.175]); global longitudinal strain (β [SE], 0.161 [0.071]); E/e’ ratio (β[SE], 0.192 [0.071]); CAC score (β [SE], 27.948 [6.116]); and log UACR (β [SE], 0.076 [0.010]). Besides, IGE was independently associated with having ≥ 2 organs involved in both overall population (OR [95% CI], 1.48 [1.23, 1.41], *P* < 0.001) and subgroups stratified by diabetes at Y25.

**Conclusion:**

Higher intensity of glycemic exposure during young adulthood was independently associated with subclinical alterations of target organs function at midlife. Our findings highlight the importance of early screening and management of IGE in youth.

## Introduction

Individuals with long-term exposure to hyperglycemia underwent chronic injuries to multiple organ systems. Previous studies have confirmed strong correlations between elevated blood glucose with coronary artery disease (CAD), diabetic cardiomyopathy, and diabetic nephropathy in late life (Devereux et al., 2000; Rossing et al., 2004; Cosentino et al., 2020). Since chronic exposure to glycemia shows detrimental impacts on target organs, a life-course evaluation of glycemic exposure considering both the magnitude and duration of exposure to hyperglycemia should be established for comprehensive assessment of its toxicity. Attempts have been made to investigate the long-term impact of glycemic exposure based on the long-term blood glucose trajectory, which was associated with mortality and cardiovascular disease (Lee et al., 2018; Ogata et al., 2019). However, long-term trajectories simply clustered the subjects into different groups based on the change tendency of blood glucose, leading to inaccurate assessment of glycemic exposure (Nagin and Odgers, 2010). Hence, the association between increased intensity of glycemic exposure (IGE) in young adulthood with target organ damages (TOD) in midlife deserves investigation.

Subclinical target organ damages of hyperglycemia, indicated by coronary artery calcium (CAC), early cardiac deformation, and dysfunction as well as albuminuria in our study, are efficient markers and well precursors of adverse clinical outcomes (BMJ, 2012; Yeboah et al., 2016; Medvedofsky et al., 2018). The Coronary Artery Risk Development in Young Adults (CARDIA) Study, which only enrolled young adults aged 18–30 years at baseline and underwent six blood glucose monitoring over 25 years, offers the opportunity to assess the impact of IGE on subclinical indicators of organ dysfunction in the life-course pattern since young adulthood.

In this study, we aimed to determine how the intensity of glycemic exposure from young adulthood to middle adulthood affects target organ function in 2,859 CARDIA study participants. We firstly assessed the associations between IGE with clinical measures of coronary artery calcification, cardiac deformation, and dysfunction as well as albuminuria. We also evaluated the association between IGE and the number of target organs involved.

## Materials and Methods

### Study Population

The datasets of the CARDIA study were obtained at the CARDIA Coordinating Center^1^ according to the application procedure required.

The CARDIA study was a prospective and observational investigation of 5,115 healthy black and white adults from 4 U.S. metropolitan communities (Birmingham, AL; Chicago, IL; Minneapolis, MN; and Oakland, CA). Briefly, men and women aged 18–30 years were recruited at 1985–1986 (Y0) and reexamined after 2 (Y2), 5 (Y5), 7 (Y7), 10 (Y10), 15 (Y15), 20 (Y20), and 25 (Y25) years. Further details of the CARDIA study have been published previously (Friedman et al., 1988). Institutional Review Boards approved the CARDIA study protocols, and all participants provided written informed consent at each examination.

For this analysis, we evaluated 3,498 participants who attended reexamination at Y25 and excluded those who had race other than white or black (*n* = 11), those with missing data on blood glucose at Y0 and Y25 (*n* = 75), and those with missing data on covariates (*n* = 553). Finally, 2,859 participants were included in our analysis. Baseline characteristics at examination in Y0 of individuals who were included and excluded are summarized in Supplementary Table 1.

### Intensity of Glycemic Exposure Assessment and Incident Diabetes

Participants were asked to fast > 8 h for measurements of fasting glucose at Y0, Y7, Y10, Y15, Y20, and Y25. Fasting glucose was measured using the hexokinase ultraviolet method by American Bio-Science Laboratories at baseline and hexokinase coupled to glucose-6-phosphate dehydrogenase at the following reexamination. Intensity of glucose exposure from young to middle adulthood was evaluated by the sum of (average blood glucose of two consecutive examinations years between the examinations) (Supplementary Figure 1). A similar approach was also used to assess the deleterious effects of elevated cumulative exposure to blood pressure in early adulthood (Kishi et al., 2015; Mahinrad et al., 2020). We determined diabetes based on any one of the following: self-report of hypoglycemic medications (measured at Y0, Y7, Y10, Y15, Y20, and Y25); fasting glucose levels ≥ 7.0 mmol/L or ≥ 126 mg/dL (measured at Y0, Y7, Y10, Y15, Y20, and Y25); 2-h post-load blood glucose ≥ 11.1 mmol/L or ≥ 200 mg/dL in 75 g oral glucose tolerance test (measured at Y10, Y20, and Y25); or glycated hemoglobin ≥ 6.5% (measured at Y20 and Y25).

### Outcome Measurements

Cardiac structure and function were assessed by echocardiographic measurements in standardized methods following American Society of Echocardiography guidelines across all field centers at Y25, as described previously (Armstrong et al., 2015; Lang et al., 2015). Echocardiographic images were collected using a commercially available cardiac ultrasound machine (Artida; Toshiba Medical Systems) by trained sonographers and were interpreted using a standard offline image analysis system (Digisonics, TX). For cardiac structure assessment, left ventricular mass (LVM) was estimated from the Devereux formula and relative wall thickness (RWT) was calculated as (diastolic interventricular septal + diastolic left ventricular [LV] posterior wall thickness)/diastolic internal LV diameter. For LV systolic function assessment, left ventricular ejection fraction (LVEF) was evaluated based on LV end-systolic and end-diastolic volumes. Global longitudinal strain (GLS), a more sensitive and prognostic index of LV systolic function, was measured using speckle-tracking echocardiography and calculated as the systolic change of segmental length divided by the segmental length at end-diastole (in percentile) (Potter and Marwick, 2018). Peak velocities in mitral inflow at early (E), late diastole (A), and early peak diastolic mitral annular velocity (e’) were measured for assessment of E/A ratio and E/e’ ratio to reflect LV diastolic function.

Coronary artery calcium (CAC) was measured by multidetector computed tomography (CT) scanner in all centers using standardized approach at Y25 (Carr et al., 2005). The analysts in CARDIA Reading Center who were blinded to participants’ information analyzed the images and calculated a CAC score using on modified Agatston method (Agatston et al., 1990). Briefly, the total CAC score in Agatston units (AU) was determined based on numbers, areas, and maximal computed tomographic numbers of the lesions. Besides, albuminuria was assessed by measurement of urine albumin to creatinine ratio (UACR). Urine albumin and urine creatinine levels were measured using nephelometry-based assay at Y25 examination according to the standardized exam protocol available at cardia.dopm.uab.edu.

For categorical analyses of significant target organ dysfunction, we defined left ventricular hypertrophy (LVH) as increased LVM > 224 g for men and > 162 g for women (Kirkpatrick et al., 2007). Concentric remodeling was defined as increased RWT > 0.42 (Lang et al., 2015). LV systolic dysfunction was defined by decreased LVEF (<50%) or abnormal GLS (>90th percentile) (Kishi et al., 2017). LV diastolic dysfunction was defined as abnormal filling patterns (E/A ratio ≤ 0.8 or ≥ 2) or increased filling pressure (E/e’ ratio > 15) (Kishi et al., 2015; Nagueh et al., 2016). We also defined CAC as an Agatston score > 0 and albuminuria as UACR > 30 mg/g.

### Covariates Measurements

Demographic characteristics, clinical history, medication use, and anthropometric and laboratory data were collected based on standardized approaches as reported in the study protocol (Friedman et al., 1988). Age, gender, race, educational attainment, and smoking and drinking status were self-reported. Smoking status and drinking status were recorded as ever/never smoking and ever/never drinking, respectively. Educational attainment was evaluated by years in school. Height and weight were measured in light clothing, and body mass index (BMI) was calculated as the ratio of weight (kg) to the square of height (m^2^). After 5 min of quiet rest, seated blood pressure (BP) in the right arm was measured three times with 1-min intervals using validated Omron HEM907XL oscillometric BP monitor at Y25 and the mean of the second and third readings was used for analysis. Plasma insulin level at Y0 and Y25 was detected via immunoassay method. Total cholesterol, high-density lipoprotein cholesterol (HDL-c) were enzymatically determined and low-density lipoprotein cholesterol (LDL-c) was calculated using the Friedewald equation.

### Statistical Analysis

Participants eligible in our analysis were subdivided into three categories based on tertiles of IGE. Baseline characteristics of participants at Y25 were compared using one-way ANOVA (continuous variables), χ^2^ tests (categorical variables), and the Kruskal-Wallis test as appropriate. Urine albumin to creatinine ratio was log-transformed due to skewed distribution. We also compared the baseline characteristics of participants in CARDIA study who were included in the final analysis with those excluded. Data were presented as means ± SD or median (interquartile range) for continuous variables and frequencies (percentages) for categorical variables.

Multivariable linear regression models were performed to evaluate the longitudinal associations between IGE over 25 years with target organs function at Y25, including cardiac structure and function, CAC, and albuminuria. Multivariable models were adjusted for covariates at Y25: model 1 included age, gender, race; model 2 was additionally adjusted for smoking, drinking, BMI, and educational attainment; model 3 was further adjusted for systolic BP, LDL-c, HDL-c, and Y25 blood glucose; and model 4 was additionally adjusted for aspirin, medication for lower cholesterol, HTN, and DM. The covariates included in the model were determined based on a *P* value < 0.05 in univariable model and clinical knowledge (Supplementary Table 2). The effects were assessed by calculating the estimates (β*s*) and standard errors (SEs) for per 1-SD increment on IGE. The powers of multivariable linear regression models based on unconditional model were shown in Supplementary Table 3. Multivariable linear regression model was performed to assess the association between IGE with insulin level and left ventricular mass index (LVMI), calculated as LVM indexed to body surface area. To investigate the robustness of the associations, we additionally adjusted for average FG across follow-up examination instead of FG in Y25 in model 5 and number of measurements of FG in model 6 in sensitivity analysis. In an explorative analysis, we further performed categorical analyses to explore the effects on significantly clinical organ dysfunction. Multivariable logistic regression models were used to evaluate the association between per 1-SD increment on IGE with target organs dysfunction, including CAC, LVH, concentric remodeling, impaired LVEF, abnormal GLS, abnormal filling patterns, increased filling pressures, and albuminuria. In logistic models, we accounted for the same covariates as in linear models and presented the effects using odds ratios (ORs) and 95% confidential intervals (95% CIs).

For further analysis in the prevalence of TOD derived from LGE, we defined numbers of TODs as the sum of the presence of cardiac deformation (LVH or concentric remodeling), cardiac systolic dysfunction (impaired LVEF or abnormal GLS), cardiac diastolic dysfunction (abnormal filling patterns or increased filling pressures), CAC, and albuminuria, ranging from 0 to 5. Multinomial logistic regression models were performed to explore the relationship between IGE and numbers of TODs (0, 1, or ≥ 2). We adjusted for traditional cardiovascular risk factors at Y25, including age, gender, race, smoking, drinking, BMI, educational attainment, SBP, LDL-c, HDL-c, Y25 blood glucose, aspirin, medication for lower cholesterol, HTN, and DM. We also compared the effects of IGE on numbers of TOD stratified by the presence of DM.

All statistical analyses were performed using SPSS version 18.0. A two-tailed *P* < 0.05 was considered statistically significant.

## Results

Baseline characteristics of 2,859 eligible participants in the CARDIA study were summarized in Table 1. Overall, the mean age at Y25 was 50.0 ± 3.6 years; 43.4% of the participants were male (*n* = 1,240) and 44.6% were black (*n* = 44.6). The average intensity of glycemic exposure was 2237.5 ± 324.7 mg/dl Yrs over 25 years in total eligible population (ranged 1717.0 to 5713.0 mg/dl Yrs). Participants with higher IGE were more frequently male, had higher BMI, SBP, DBP, fasting glucose at Y0 and Y25 (all *P* < 0.001). Among them, only very few participants (*n* = 10, 0.3%) had diabetes at Y0 and 373 participants (13.0%) developed diabetes up to Y25. Supplementary Table 4 described the baseline characteristics of participants who had diabetes and remained free from diabetes at Y25. Participants who developed diabetes were more likely black and had higher IGE, BMI, SBP, and DBP.

**TABLE 1:** Baseline Characteristics for 2,859 CARDIA participants.

**Characteristic**	**Total (*N* = 2,859)**	**Intensity of Glycemic exposure**			
		**Group 1 (*N* = 953)**	**Group 2 (*N* = 956)**	**Group 3 (*N* = 950)**	***P*-value**
IGE (mg/dl Yrs)	2237.5 ± 324.7	2016.4 ± 72.2	2183.2 ± 42.1	2514.0 ± 426.5	<0.001
Age, years	50.0 ± 3.6	49.4 ± 3.7	50.0 ± 3.6	50.6 ± 3.4	<0.001
Male	1240 (43.4)	213 (22.4)	442 (46.2)	585 (61.6)	<0.001
Black	1275 (44.6)	436 (45.8)	382 (40.0)	457 (48.1)	0.001
BMI, kg/m2	29.9 ± 6.8	27.7 ± 6.2	29.8 ± 6.5	32.2 ± 6.8	<0.001
SBP, mmHg	118.2 ± 15.3	115.3 ± 15.3	118.1 ± 14.7	121.3 ± 15.2	<0.001
DBP, mmHg	73.6 ± 10.8	71.6 ± 11.1	73.7 ± 10.5	75.4 ± 10.4	<0.001
FG at Y0, mg/dl	81.9 ± 10.4	76.6 ± 6.0	81.8 ± 7.4	87.2 ± 13.2	<0.001
FG at Y25, mg/dl	98.6 ± 27.0	86.5 ± 6.6	94.0 ± 7.6	115.3 ± 40.5	<0.001
Insulin at Y0, uU/mL	10.4 ± 7.2	8.9 ± 5.8	9.8 ± 6.0	12.3 ± 9.0	<0.001
Insulin at Y25, uU/mL	11.0 ± 9.7	8.0 ± 5.7	10.7 ± 7.8	14.4 ± 12.9	<0.001
DM at Y0	10 (0.3%)	0	3 (0.3)	7 (0.7)	0.011
DM at Y25	373 (13.0%)	20 (2.1)	57 (6.0)	296 (31.2)	<0.001
Smoking	1541 (53.9)	491 (51.5)	512 (53.6)	538 (56.6)	0.079
Drinking	2253 (78.8)	750 (78.7)	772 (80.8)	731 (76.9)	0.126
Educational attainment	14.8 ± 1.8	14.9 ± 1.8	14.9 ± 1.8	14.5 ± 1.9	<0.001
LDL-c, mg/dl	111.8 ± 32.6	109.4 ± 30.5	113.9 ± 31.8	112.0 ± 35.3	0.011
HDL-c, mg/dl	58.5 ± 18.0	65.2 ± 18.4	57.8 ± 17.4	52.6 ± 15.7	<0.001
DM medication	201 (7.0)	7 (0.7)	16 (1.7)	178 (18.7)	<0.001
HTN medication	761 (26.6)	164 (17.2)	236 (24.7)	361 (38.0)	<0.001
Lipid medication	437 (15.3)	79 (8.3)	117 (12.2)	241 (25.4)	<0.001
Aspirin use	480 (16.8)	97 (10.2)	146 (15.3)	237 (24.9)	<0.001
**Echocardiographic parameters**					
LVM (*n* = 2553)	167.3 ± 51.9	148.5 ± 44.4	168.8 ± 48.5	186.8 ± 55.5	<0.001
RWT (*n* = 2549)	0.4 ± 0.1	0.3 ± 0.1	0.4 ± 0.1	0.4 ± 0.1	0.005
LVEF (*n* = 2553)	69.8 ± 8.0	69.9 ± 7.4	69.9 ± 7.8	69.6 ± 8.8	0.773
GLS (*n* = 2497)	−15.1 ± 2.4	−15.2 ± 2.3	−15.2 ± 2.3	−14.5 ± 2.5	<0.001
E/A (*n* = 2810)	1.3 ± 0.4	1.4 ± 0.4	1.3 ± 0.3	1.2 ± 0.4	<0.001
E/e’ (*n* = 2785)	9.0 ± 2.7	8.8 ± 2.6	8.8 ± 2.7	9.3 ± 2.9	<0.001
CAC score, AU (*n* = 2616)	0.0 (0.0, 3.9)	0.0 (0.0, 0.0)	0.0 (0.0, 1.9)	0.0 (0.0, 29.0)	<0.001
UACR, mg/g (*n* = 2794)	4.7 (3.3, 8.1)	4.7 (3.3, 7.5)	4.4 (3.1, 7.1)	5.1 (3.4, 10.2)	<0.001

*Data are presented as mean ± SD, number (percentage), or median (interquartile range), as appropriate. Sample characteristics were drawn from examination 8 at Y25 unless otherwise indicated. IGE, intensity of glycemic exposure; BMI, body mass index; SBP, systolic blood pressure; DBP, diastolic blood pressure; FG, fasting glucose; DM, diabetes mellitus; HDL-C, high-density lipoprotein cholesterol; LDL-C, low-density lipoprotein cholesterol; CAC, coronary artery calcium; LVM, left ventricular mass; RWT, relative wall thickness; LVEF, left ventricular ejection fraction; GLS, global longitudinal peak strain; UACR, urine albumin to creatinine ratio.*

### IGE and Target Organ Function

**FIGURE 1:**
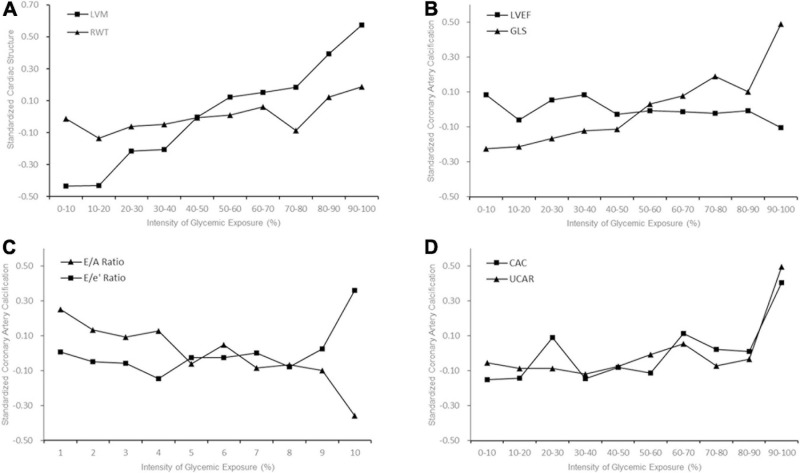
Early Adulthood Intensity of Glycemic Exposure and Middle-Age Target Organ Function. The trajectory slopes showed indices of target organs function with increasing decile of intensity of glycemic exposure (IGE). **(A)** Cardiac structure; **(B)** Left ventricular (LV) systolic function; **(C)** LV diastolic function; **(D)** Coronary artery calcium (CAC) and albuminuria. Higher deciles of IGE produced higher level of left ventricular mass (LVM) **(A)**, global longitudinal strain (GLS), CAC and urine albumin to creatinine ratio (UACR).

As shown in Figure 1, higher deciles of IGE produced higher LVM, GLS, E/e’ ratio, CAC score, UACR, and lower E/A ratio. Table 2 represented the associations between IGE over 25 years with target organs function parameters in linear regression models. In model 1, a 1-SD-increment higher IGE was significantly associated with higher LVM, E/e’ ratio, CAC score, log UACR, and lower E/A (all *P* < 0.001). Further adjustments for smoking, drinking, BMI, and educational attainment in model 2 did not alter the results. The association between E/A ratio with IGE dissipated after further adjustments in model 3, while other associations remained significant. A 1-SD increment of IGE were still positively associated with LVM (β [SE], 5.468 [1.175]), GLS (β [SE], 0.161 [0.071]), E/e’ ratio (β[SE], 0.192 [0.071]), CAC score (β [SE], 27.948 [6.116]), and log UACR (β [SE], 0.076 [0.010]) after full adjustment in model 4. IGE over 25 years was positively associated with serum insulin and LVMI at Y25 (Supplementary Table 5). Sensitivity analysis with additional adjustment for average blood glucose in model 5 and number of measurements of FG in model 6 demonstrated consistent results (Supplementary Table 6).

**TABLE 2:** Linear regression models to examine the associations between intensity of glycemic exposure during young adulthood with target organ function in midlife.

**Variables**	**Per 1 SD increment of IGE (standardized value)**			
	**Model 1**	**Model 2**	**Model 3**	**Model 4**
	
	**β (SE)**	**β (SE)**	**β (SE)**	**β (SE)**
**Cardiac structure and function**				
**Structure**				
LVM (*n* = 2553)	8.359(0.890)***	4.139(0.829)***	5.293(1.085)***	5.468(1.175)***
RWT (*n* = 2549)	0.002(0.001)	0.000(0.001)	−0.001(0.002)	−0.002(0.002)
**Systolic Function**				
LVEF (*n* = 2553)	−0.071(0.157)	−0.075(0.161)	−0.100(0.215)	−0.024(0.233)
GLS (*n* = 2497)	0.398(0.049)***	0.318(0.050)***	0.214(0.067)**	0.161(0.071)*
**Diastolic Function**				
E/A (*n* = 2810)	−0.048(0.007)***	−0.030(0.007)***	−0.017(0.009)	−0.007(0.010)
E/e’ (*n* = 2785)	0.367(0.051)***	0.260(0.051)***	0.207(0.066)**	0.192(0.071)**
**Subclinical Atherosclerosis**				
CAC score (*n* = 2616)	35.315(4.303)***	34.917(4.393)***	30.161(5.707)***	27.948(6.116)***
**Renal Function**				
Log UACR (*n* = 2794)	0.115(0.007)***	0.111(0.007)***	0.085(0.010)***	0.076(0.010)***

IGE, intensity of glycemic exposure. All models were adjusted for covariates measured at Y25. Model 1 was adjusted for age, gender, race; Model 2 was additionally adjusted for smoking, drinking, BMI, educational attainment; Model 3 was additionally adjusted for SBP, LDL-c, HDL-c, Y25 blood glucose; Model 4 was additionally adjusted for aspirin, medication for lower cholesterol, HTN, and DM. ***P < 0.001; **P < 0.01; *P < 0.05.

Supplementary Table 7 presented multivariable logistic regression models to further investigate the impacts of IGE on subclinical target organ dysfunction. For cardiac structural and functional outcomes, per 1-SD increment of IGE was significantly associated with a higher prevalence of LVH (OR [95% CI], 1.25 [1.09, 1.43]) and abnormal GLS (OR [95% CI], 1.22 [1.02, 1.45]) in a fully adjusted model. In contrast, IGE was not related to clinical diastolic dysfunction, including abnormal filling patterns and increased filling pressure after full adjustments. Besides, per 1-SD increment of IGE showed correlations with higher prevalence of subclinical atherosclerosis (OR [95% CI], 1.24 [1.08, 1.43]) and albuminuria (OR [95% CI], 1.40 [1.20, 1.63]).

### IGE and Number of TOD

**FIGURE 2:**
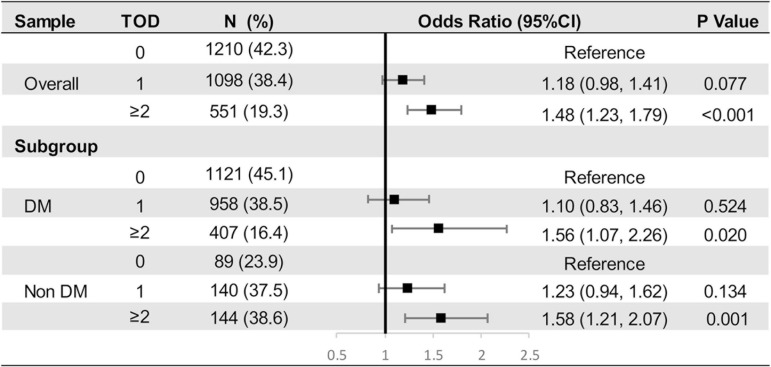
Multinomial Logistic Regression Models to Examine the Odds Ratio of Having 0, 1 or ≥ 2 Target Organ Damages by Intensity of Glycemic Exposure.

Prevalence of TOD (0, 1, or ≥ 2 organs damaged) in the overall population and subgroups stratified by incident diabetes were shown in Figure 2. Compared with the non-diabetes population, those with DM at Y25 had a higher prevalence of 2 or more damaged organs, indicating a greater vulnerability to target organ damages in the diabetes population. Figure 2 presented the odds ratio of having 1 or ≥ 2 organs damaged by long-term IGE in multivariable multinomial logistic regression models. A 1-SD increment of IGE was independently associated with having ≥ 2 organs involved in overall population (OR [95% CI], 1.48 [1.23, 1.41]; *P* < 0.001; Figure 2), suggesting the higher the levels of IGE, the more advanced the target organs involvement. It should be noted that the association were statistically significant in both DM and non-DM subgroups.

## Discussion

In this prospective cohort of young adults followed up over 25 years, we demonstrated greater IGE during young adulthood was associated with unfavorable impairment of multiple target organs, including subclinical cardiac structural and functional impairment, subclinical atherosclerosis, and albuminuria independent of fasting glucose at Y25. The long-term IGE was associated with the prevalence of TOD and the higher IGE tended to involve more target organs, which remained consistent even among those free from diabetes at Y25.

Previous researches have established that diabetes is responsible for multiple target organs injury (De Simone et al., 2017). Diabetic cardiomyopathy is characterized by myocardial hypertrophy and myocardial remodeling, manifested as diastolic dysfunction in the earlier and systolic dysfunction during disease progression (Boudina and Abel, 2007). Besides, individuals with diabetes are also at increased risk of atherosclerosis and diabetic nephropathy in later life (Rossing et al., 2004; Raffield et al., 2018). Overall, these prior studies suggested that diabetes is a strong predictor of adverse clinical events in later life. However, previous studies always investigated the detrimental impacts of hyperglycemia based on measurement of blood glucose at a single time point and whether lifespan exposure to hyperglycemia derived subclinical adverse effects earlier is still unknown. To the best of our knowledge, our study was the first to comprehensively focus on the chronic impacts of the long-term intensity of glycemic exposure during young adulthood on multiple target organs in midlife, accounting for the magnitude and duration of glycemic exposure simultaneously. Our findings extended the prior studies by showing that higher IGE over the 2nd, 3rd, and 4th decade of life was associated with earlier target organs dysfunction at the 5th decade. Besides, our results also suggested that the numbers of target organs involved depended on the cumulative effects of IGE, even in a non-diabetic subset of participants who were younger with fewer clinical comorbidities.

Prior to our study, there have been several metrics concerning the effect of long-term glycemic exposure. Visit-to-visit FG variability in young adulthood was correlated with incident cardiovascular disease and all-cause mortality in the CARDIA study (Bancks et al., 2019b). Visit-to-visit FG variability mediates the effect of long-term glycemic exposure in part; however, it does not capture the impact derived from the magnitude of glycemic exposure. Duration of diabetes, associated with atherosclerosis and adverse outcome, was a surrogate measurement of lifespan glycemia exposure but was not available for subclinical hyperglycemia assessment (Fox et al., 2004; Kim et al., 2015). Besides, based on group-based trajectory modeling, participants with three or more FG measurements were clustered into distinct groups, each with a specific trajectory (Nagin and Odgers, 2010; Yuan et al., 2018). Bancks et al. (2019a) developed trajectory curves to visualize the long-term FG tendency in the CARDIA study, observing impaired LV structure and diastolic function in both moderate-increasing and high-stable FG trajectory groups compared to the low-stable FG group. Nevertheless, the classification for distinctive groups was roughly based on the FG tendency and not precise enough for clinical assessment (Jennings and Muniz, 2016). In trajectory methodology, the grouping is automatically assumed to exist and the findings were data-driven, so the conclusion needs to be interpreted with great caution (Jennings and Muniz, 2016). In the present study, IGE seems to be a more concrete and accurate indicator for long-term glycemic exposure assessment.

The underlying pathophysiologic pathways that link higher long-term IGE with target organs impairment may share common pathogenic mechanisms. Studies have shown that hyperglycemia induces and accelerates the deposition and accumulation of glycosylation products within myocardium and arterial wall, leading to cardiac and vascular injury (Naka et al., 2004; Boudina and Abel, 2007; West et al., 2009). Besides, endothelial dysfunction is also a main pathogenic factor contributing to diabetic vascular impairment, e.g., initiating the pathogenesis of atherosclerosis (Calles-Escandon and Cipolla, 2001). Furthermore, hyperglycemia and insulin resistance also promote myocardial collagen deposition and myocyte hypertrophy, causing myocardial remodeling (Kishi et al., 2017). Some other mechanisms may also involve the pathogenesis, including oxidative stress, altered substrate metabolism, mitochondrial dysfunction, inflammation activation, and so on (Boudina and Abel, 2007; Ceriello and Testa, 2009; Donath and Shoelson, 2011).

For cardiac functional measurements, it has been demonstrated that global longitudinal strain is a sensitive marker of systolic dysfunction than ejection fraction and provides additional prognostic value on adverse outcomes (Russo et al., 2014; Potter and Marwick, 2018). Meanwhile, previous studies indicated that E/e’ ratio was more predictive for primary cardiac events than E/A ratio (Sharp et al., 2010). In our study, we noted associations between long-term IGE with worse global longitudinal strain but not ejection fraction. We also found that IGE over young adulthood was associated with a worse E/e’ ratio rather than E/A. Therefore, GLS and E/e’ ratio seemed to be powerful in showing the adverse effects of IGE that classical echocardiographic indicators failed to identify, which may be explained by a young age of CARDIA population with a lower prevalence of clinical organ dysfunction at advanced stage. Similarly, IGE was also associated with CAC and albuminuria, the well-known and powerful precursors of adverse outcomes (Detrano et al., 2008; BMJ, 2012). In fact, identification of the predisposing factors for subclinical organ dysfunction provides insight toward the long-term risk of organ dysfunction. From a disease-prevention perspective, given the independent prognostic significance of TOD, it would make sense to establish screening strategies on IGE and implement intensive blood glucose management during young adulthood, avoiding irreversible organ damage (BMJ, 2012; Yeboah et al., 2016; Medvedofsky et al., 2018).

An unexpected finding was that long-term IGE showed subclinical adverse effects on target organs in both diabetes and non-diabetes subgroups, which highlights the need to investigate the cumulative effects of glycemic exposure in participants below the diagnostic criteria of diabetes. Indeed, even in examination at Y25, only a minority of participants (13.0%) were diagnosed as diabetes and the FG level in the non-diabetes population was on average below the current threshold of prediabetes (92.8 ± 9.1 mg/dl, Supplementary Table 4), suggesting exposure to subclinical hyperglycemia over young adulthood could precipitate organ dysfunction. A prospective study may be warranted to determine the optimal cut-off points for evaluation of the subclinical impacts of cumulative glycemic exposure. On the other hand, given the subclinical hyperglycemia is a modifiable factor, the optimal glycemic control target remains controversial despite several randomized controlled trials (RCTs) published (UK Prospective Diabetes Study (UKPDS) Group, 1998; Gerstein et al., 2008; Holman et al., 2008; Patel et al., 2008). UKPDS trial demonstrated a great microvascular benefit from intensive glucose control therapy, which was confirmed in subsequent ADVANCE and ACCORD trials. However, whether intensive glucose control therapy reduces the risk of macrovascular events is still disputed. Indeed, the participants in these RCTs were characterized by old age, long duration of diabetes, and poor glycemic control. Irreversible subclinical organ impairment in these participants may weaken the effects of intensive glucose control therapy and lead to unclear macrovascular benefit. Thus, further research is needed to clarify whether intensive glucose control in young patients with subclinical hyperglycemia can prevent or delay the early organ dysfunction, ultimately improving prognosis. Nevertheless, the accompanying risk of adverse reaction, especially hypoglycemic episodes, should be carefully assessed.

Our study results are strengthened by a population-based cohort with a large sample size; regular screening fasting glucose using standardized protocols over 25 years, facilitating assessment of the cumulative intensity of glycemic exposure; a cohort of young adults with fewer comorbidities, almost non-diabetic population, was optimal for exploring the subclinical effects of IGE on target organs and providing evidence on prevention of diabetes-induced end-organ damage. However, several limitations in our study need to be considered. First, cardiac function and CAC were measured in detail until examination in Y25, hindering further assessment of the long-term changes of target organs function from young adulthood to midlife. Hence, we can only assume that the measurements in Y25 could reflect the decline in organ function. Second, only approximately half of participants in CARDIA performed echocardiographic measurements and CAC assessment in Y25, thus our analysis may be subjected to selection bias. To address this issue, we further compared the baseline characteristics between the analyzed sample with the excluded sample (Supplementary Table 1). The participants who were excluded in our study were more frequently male, black, smokers, have lower educational attainment, and higher FG and SBP levels. In fact, the participants included in our analysis may have a lower risk of target organs impairment than those excluded, which likely creates bias toward the null and may underestimate the deleterious effects of IGE. Third, owing to the calculation method of IGE, each individual had unequal number of measurements of FG, and those who were less measured may have inaccurate evaluation of glycemic exposure. Indeed, more than 80% of individuals completed all 6 measurements of FG and we observed consistent associations when additionally adjusted for number of measurements (Supplementary Table 6). Fourth, given the nature of the observational study, our findings may be susceptible to reverse causation and residual confounding. Last but not least, CARDIA is a biracial cohort including white and black individuals, so that our findings required external validation in other ethnic populations, e.g., Asians.

## Conclusion

In conclusion, our study finds associations between the long-term intensity of glycemic exposure during young adulthood with subclinical impairment of cardiac structure and function, CAC, and albuminuria at midlife. Higher IGE is also associated with increased numbers of target organs involvement, even in non-diabetic populations. Our findings emphasize the importance of screening and management of subclinical hyperglycemia in youth, thus preventing or delaying the early organ dysfunction and ultimately improving the prognosis.

## Data Availability Statement

The raw data supporting the conclusions of this article will be made available by the authors, without undue reservation.

## Ethics Statement

Ethical review and approval was not required for the study on human participants in accordance with the local legislation and institutional requirements. The patients/participants provided their written informed consent to participate in this study.

## Author Contributions

XL and XZhu conceived and designed the study, obtained funding, and acquired the data. YiL and XZho conceived and designed the study, performed all analysis and interpretation of data, and drafted the manuscript. ZX, SZ, and ML advised on statistical analysis methods and critically revised the manuscript for important content. YF, YH, and XS interpreted the data and critically revised the manuscript for important content. HZ, XX, YG, YuL, DY, and XY critically revised the manuscript for important content and contributed to the discussion. XL was the guarantor of this work and, as such, had full access to all the data in the study and took responsibility for the integrity of the data and the accuracy of the data analysis. All authors contributed to the article and approved the submitted version.

## Conflict of Interest

The authors declare that the research was conducted in the absence of any commercial or financial relationships that could be construed as a potential conflict of interest.

## Footnotes

1 https://www.cardia.dopm.uab.edu/
