# Supplementary material for: Intensity of Glycemic Exposure in Early Adulthood and Target Organ Damage in Middle Age: The CARDIA Study
Source: Front Physiol. 2021 Jun 23;12:614532. doi: 10.3389/fphys.2021.614532 (PMC8260980; doi:10.3389/fphys.2021.614532)
Supplement: Supplementary file 1 [file Table_1.DOCX]

**Supplemental materials**

**Supplemental Figure 1 One Example Showing the Calculation of Intensity of Glycemic Exposure across 6 Visits.**

**Supplemental Table 1 Baseline Characteristics at Y0 Examination of Participants Who Were Included and Excluded in Our Analysis**

**Supplemental Table 2 Univariable Linear Regression Models Showed the Associations between Covariates with Target Organ Function**

**Supplemental Table 3 The Powers in Multivariable Linear Regression Models Based on Unconditional Model**

**Supplemental Table 4 Baseline Characteristics at Y25 Examination of 2,859 CARDIA Participants, Stratified by Diabetes**

**Supplemental Table 5 Multivariable Linear Regression Models Showed the Associations between Covariates with Serum Insulin and LVMI**

**Supplemental Table 6 Sensitivity Analysis Showing the Associations between Intensity of Glycemic Exposure During Young Adulthood with Target Organ Function in Midlife**

**Supplemental Table 7 Logistic Regression Models to Examine the Associations Between Intensity of Glycemic Exposure During Young Adulthood with Target Organ Damage in Midlife**

**Figure legend**

**Supplemental Figure 1 One Example Showing the Calculation of Intensity of Glycemic Exposure across 6 Visits.**

A1-A5 represented the mean FG of two successive FG measurements. Intensity of glycemic exposure was calculated as $(A1\times7 years+A2\times3 years+A3\times5 years+A4\times5 years+A5\times5 years)$, shown by the shadow area, mg/dl$\times$years.

**Supplemental Table 1 Baseline Characteristics at Y0 Examination of Participants Who Were Included and Excluded in Our Analysis**

| Characteristic | Participants Included  (N=2859) | Participants Excluded  (N=373) | P value |
| --- | --- | --- | --- |
| Age, years | 25.0±3.6 | 24.4±3.7 | <0.001 |
| Male | 1240 (43.4) | 1088 (48.3) | <0.001 |
| Black | 1275 (44.6) | 1367 (60.6) | <0.001 |
| BMI, kg/m^2^ | 24.3±4.6 | 24.6±5.0 | 0.107 |
| SBP, mmHg | 109.9±10.7 | 111.1±11.2 | <0.001 |
| DBP, mmHg | 68.4±9.4 | 68.9±9.9 | 0.061 |
| FG, mg/dl | 81.9±10.4 | 83.6±21.7 | <0.001 |
| Smoking | 1623 (56.8) | 1419 (63.0) | <0.001 |
| Drinking | 2472 (86.6) | 1931 (85.9) | 0.396 |
| Educational attainment | 14.2±3.5 | 13.7±5.8 | <0.001 |
| LDL-c, mg/dl | 111.8±32.6 | 111.8±32.6 | 0.102 |
| HDL-c, mg/dl | 53.4±12.8 | 52.9±13.8 | 0.170 |

Data are presented as mean ± SD or number (percentage), as appropriate.

BMI, body mass index; SBP, systolic blood pressure; DBP, diastolic blood pressure; FG, fasting glucose; DM, diabetes mellitus; HDL-C, high-density lipoprotein cholesterol; LDL-C, low-density lipoprotein cholesterol.

**Supplemental Table 2 Univariable Linear Regression Models Showed the Associations between Covariates with Target Organ Function**

| Covariates | LVM | | RWT | | LVEF | | GLS | | E/A |  | E/e’ |  | CAC |  | LOG UACR | |
| --- | --- | --- | --- | --- | --- | --- | --- | --- | --- | --- | --- | --- | --- | --- | --- | --- |
|  | β (SE) | P value | β (SE) | P value | β (SE) | P value | β (SE) | P value | β (SE) | P value | β (SE) | P value | β (SE) | P value | β (SE) | P value |
| Age, years | 0.415  (0.285) | 0.15 | -0.000  (0.000) | 0.77 | 0.142  (0.044) | 0.001 | -0.005  (0.014) | 0.70 | -0.015  (0.002) | <  0.001 | 0.063  (0.014) | <  0.001 | 6.925  (1.997) | <  0.001 | 0.003  (0.002) | 0.111 |
| Gender | -44.518  (1.885) | <  0.001 | -0.008  (0.003) | 0.007 | 1.846  (0.318) | <0.001 | -0.783  (0.096) | <  0.001 | -0.036  (0.014) | 0.009 | 0.448  (0.104) | <  0.001 | -62.256  (8.636) | <  0.001 | 0.085  (0.015) | <  0.001 |
| Race | -18.396  (2.031) | <  0.001 | -0.034  (0.003) | <0.001 | -0.197  (0.317) | 0.54 | -0.996  (0.096) | <  0.001 | 0.070  (0.014) | <  0.001 | -0.950  (0.102) | <  0.001 | 11.473  (8.679) | 0.19 | -0.122  (0.015) | <  0.001 |
| BMI, kg/m2  (per 1 SD) | 21.218  (0.973) | <  0.001 | 0.012  (0.001) | <0.001 | 0.108  (0.163) | 0.51 | 0.539  (0.050) | <  0.001 | -0.082  (0.007) | <  0.001 | 0.572  (0.051) | <  0.001 | 1.925  (4.343) | <  0.001 | 0.058  (0.007) | <  0.001 |
| SBP, mmHg  (per 1 SD) | 16.641  (0.980) | <  0.001 | 0.013  (0.001) | <0.001 | 0.147  (0.159) | 0.36 | 0.553  (0.048) | <  0.001 | -0.079  (0.007) | <  0.001 | 0.712  (0.050) | <  0.001 | 15.692  (4.336) | <  0.001 | 0098  (0.007) | <  0.001 |
| FG at Y25, mg/dl (per 1 SD) | 9.672  (1.014) | <  0.001 | 0.004  (0.001) | 0.003 | -0.157  (0.158) | 0.32 | 0.422  (0.048) | <  0.001 | -0.048  (0.007) | <  0.001 | 0.286  (0.051) | <  0.001 | 30.501  (4.226) | <  0.001 | 0.093  (0.007) | <  0.001 |
| Smoking | 7.102  (2.053) | <  0.001 | 0.003  (0.003) | 0.29 | 0.117  (0.316) | 0.71 | -0.032  (0.097) | 0.74 | -0.025  (0.014) | 0.07 | 0.153  (0.103) | 0.14 | 16.567  (8.665) | 0.006 | 0.008  (0.015) | 0.60 |
| Drinking | -5.356  (2.528) | 0.03 | -0.008  (0.003) | 0.02 | 0.170  (0.389) | 0.66 | -0.504  (0.121) | <  0.001 | 0.031  (0.017) | 0.07 | -0.881  (0.125) | <  0.001 | -3.562  (10.565) | 0.74 | -0.027  (0.018) | 0.14 |
| Education, years | -4.047  (0.552) | <  0.001 | -0.006  (0.001) | <  0.001 | 0.031  (0.086) | 0.72 | -0.162  (0.026) | <  0.001 | 0.024  (0.004) | <  0.001 | -0.209  (0.028) | <  0.001 | -3.710  (2.336) | 0.11 | -0.023  (0.004) | <  0.001 |
| LDL-c, mg/dl  (per 1 SD) | -1.766  (1.032) | 0.09 | -0.001  (0.001) | 0.32 | -0.162  (0.159) | 0.31 | 0.032  (0.049) | 0.50 | -0.013  (0.007) | 0.05 | -0.105  (0.052) | 0.043 | -1.903  (4.295) | 0.66 | -0.022  (0.007) | 0.003 |
| HDL-c, mg/dl  (per 1 SD) | -14.666  (0.973) | <  0.001 | -0.007  (0.001) | <  0.001 | 0.286  (0.156) | 0.07 | -0.504  (0.047) | <  0.001 | 0.030  (0.007) | <  0.001 | -0.216  (0.052) | <  0.001 | -6.986  (4.325) | 0.11 | -0.005  (0.007) | 0.50 |
| DM medication | 30.036  (7.327) | <  0.001 | 0.020  (0.006) | <0.001 | -0.569  (0.636) | 0.37 | 1.579  (0.198) | <  0.001 | -0.193  (0.027) | <  0.001 | 1.260  (0.202) | <  0.001 | 79.700  (16.623) | <  0.001 | 0.344  (0.028) | <  0.001 |
| HTN medication | 26.322  (2.296) | <  0.001 | 0.025  (0.003) | <0.001 | 0.807  (0.361) | 0.26 | 0.968  (0.111) | <  0.001 | -0.149  (0.015) | <  0.001 | 1.087  (0.115) | <  0.001 | 73.700  (9.643) | <  0.001 | 0.173  (0.016) | <  0.001 |
| Lipid medication | 16.910  (2.876) | <0.001 | 0.017  (0.004) | <0.001 | 0.551  (0.444) | 0.22 | 0.627  (0.136) | <  0.001 | -0.110  (0.019) | <  0.001 | 0.730  (0.142) | <  0.001 | 100.073  (11.824) | <  0.001 | 0.109  (0.020) | <  0.001 |
| Aspirin use | 17.670  (2.770) | <0.001 | 0.013  (0.004) | <0.001 | -0.126  (0.429) | 0.77 | 0.696  (0.131) | <  0.001 | -0.089  (0.018) | <  0.001 | 0.606  (0.138) | <  0.001 | 65.827  (11.442) | <  0.001 | 0.052  (0.020) | 0.008 |

IGE, intensity of glycemic exposure.

All models were adjusted for age, gender, race, smoking, drinking, BMI, educational attainment, SBP, LDL-c, HDL-c, Y25 blood glucose, aspirin, medication for lower cholest, HTN and DM.

**Supplemental Table 3 The Powers in Multivariable Linear Regression Models Based on Unconditional Model[1; 2; 3; 4]**

|  | N | Variables controlled | variables tested | Alpha | Beta | ρ0² | ρ1² | Power |
| --- | --- | --- | --- | --- | --- | --- | --- | --- |
| LVM | 2553 | 15 | 1 | 0.05 | 0.0071 | 0.20 | 0.26 | 0.9929 |
| RWT | 2549 | 15 | 1 | 0.05 | 0.0071 | 0.20 | 0.26 | 0.9929 |
| LVEF | 2553 | 15 | 1 | 0.05 | 0.0071 | 0.20 | 0.26 | 0.9929 |
| GLS | 2497 | 15 | 1 | 0.05 | 0.0080 | 0.20 | 0.26 | 0.9920 |
| E/A | 2810 | 15 | 1 | 0.05 | 0.0040 | 0.20 | 0.26 | 0.9960 |
| E/e’ | 2785 | 15 | 1 | 0.05 | 0.0042 | 0.20 | 0.26 | 0.9958 |
| CAC score | 2616 | 15 | 1 | 0.05 | 0.0061 | 0.20 | 0.26 | 0.9939 |
| Log UACR | 2794 | 15 | 1 | 0.05 | 0.0041 | 0.20 | 0.02 | 0.9959 |

**Supplemental Table 4 Baseline Characteristics at Y25 Examination of 2,859 CARDIA Participants, Stratified by Diabetes**

| Characteristic | Diabetes  (N=373) | Free from diabetes  (N=2486) | P  value |
| --- | --- | --- | --- |
| IGE (mg/dl$\times Yrs$) | 2709.2±633.0 | 2166.7±151.2 | <0.001 |
| Age, years | 50.5±3.6 | 50.0±3.6 | 0.016 |
| Male | 163 (43.7) | 1077 (43.3) | 0.891 |
| Black | 253 (67.8) | 1022 (41.1) | <0.001 |
| BMI, kg/m2 | 34.7±7.2 | 29.1±6.4 | <0.001 |
| SBP, mmHg | 123.7±17.0 | 117.4±14.8 | <0.001 |
| DBP, mmHg | 76.7±10.7 | 73.1±10.7 | <0.001 |
| FG at Y0, mg/dl | 86.5±20.2 | 81.2±7.8 | <0.001 |
| FG at Y25, mg/dl | 137.0±57.7 | 92.8±9.1 | <0.001 |
| DM at Y0 | 10 (0.3) | 0 (0) | <0.001 |
| Smoking | 212 (56.8) | 1329 (53.5) | 0.222 |
| Drinking | 249 (66.8) | 2004 (80.6) | <0.001 |
| Educational attainment | 14.1±1.8 | 14.9±1.8 | <0.001 |
| LDL-c, mg/dl | 105.1±36.1 | 112.8±32.0 | <0.001 |
| HDL-c, mg/dl | 51.9±16.6 | 59.5±17.9 | <0.001 |
| DM medication | 201 (7.0) | 0 (0) | <0.001 |
| HTN medication | 234 (62.7) | 527 (21.2) | <0.001 |
| Lipid medication | 160 (42.9) | 277(11.1) | <0.001 |
| Aspirin use | 143 (38.3) | 337 (13.6) | <0.001 |
| Echocardiographic parameters | | | |
| LVM (n=2553) | 189.6±61.7 | 164.1±49.5 | <0.001 |
| RWT (n=2549) | 0.4±0.1 | 0.4±0.1 | <0.001 |
| LVEF (n=2553) | 69.5±10.0 | 69.9±7.6 | 0.417 |
| GLS (n=2497) | -14.1±2.7 | -15.3±2.4 | <0.001 |
| E/A (n=2810) | 1.1±0.3 | 1.3±0.4 | <0.001 |
| E/e’ (n=2785) | 10.3±3.0 | 8.8±2.6 | <0.001 |
| CAC score, AU (n=2616) | 0.0 (0.0, 34.9) | 0.0 (0.0, 1.9) | <0.001 |
| UACR, mg/g (n=2794) | 7.8 (4.5, 18.9) | 4.5 (3.2, 7.2) | <0.001 |

Data are presented as mean±SD, N (percentage) or median (interquartile range), as appropriate.

IGE, intensity of glycemic exposure; BMI, body mass index; SBP, systolic blood pressure; DBP, diastolic blood pressure; FG, fasting glucose; DM, diabetes mellitus; TG, triglyceride; HDL-C, high-density lipoprotein cholesterol; LDL-C, low-density lipoprotein cholesterol; CAC, coronary artery calcium; LVM, left ventricular mass; RWT, relative wall thickness; LVEF, left ventricular ejection fraction; GLS, global longitudinal peak strain; UACR, urine albumin to creatinine ratio.

**Supplemental Table 5 Multivariable Linear Regression Models Showed the Associations between Covariates with Serum Insulin and LVMI**

| Variables | Per 1 SD increment of IGE (standardized value) | | | |
| --- | --- | --- | --- | --- |
|  | Model 1 | Model 2 | Model 3 | Model 4 |
|  | β (SE) | β (SE) | β (SE) | β (SE) |
| LVMI (n=2553) | 2.110 (0.389) ^***^ | 1.749 (0.396) ^***^ | 2.430 (0.517) ^***^ | 2.454 (0.560) ^***^ |
| Insulin at Y25,  uU/ML (n=2844) | 2.335 (0.179) ^***^ | 1.313 (0.165) ^***^ | 0.908 (0.217) ^***^ | 0.522 (0.231) ^*^ |

IGE, intensity of glycemic exposure; LVMI, left ventricular mass index.

All models were adjusted for covariates measured at Y25.

Model 1 was adjusted for age, gender, race; Model 2 was additionally adjusted for smoking, drinking, BMI, educational attainment; Model 3 was additionally adjusted for SBP, LDL-c, HDL-c, Y25 blood glucose; Model 4 was additionally adjusted for aspirin, medication for lower cholest, HTN and DM.

***, p <0.001; **, p <0.01; *, p <0.05.

**Supplemental Table 6 Sensitivity Analysis Showing the Associations between Intensity of Glycemic Exposure During Young Adulthood with Target Organ Function in Midlife**

| Variables | Per 1 SD increment of IGE (standardized value) | | | |
| --- | --- | --- | --- | --- |
|  | Model 5 | | Model 6 | |
|  | β (SE) | P value | β (SE) | P value |
| Cardiac Structure and Function | | | | |
| Structure | | | | |
| LVM (n=2553) | 5.213 (1.302) ^***^ | <0.001 | 5.449 (1.175) ^***^ | <0.001 |
| RWT (n=2549) | 0.002 (0.001) | 0.389 | -0.001 (0.002) | 0.464 |
| Systolic Function | | | | |
| LVEF (n=2553) | -0.078 (0.258) | 0.762 | -0.025 (0.233) | 0.916 |
| GLS (n=2497) | 0.193 (0.078) ^*^ | 0.014 | 0.162 (0.071) ^*^ | 0.023 |
| Diastolic Function | | | | |
| E/A (n=2810) | -0.006 (0.011) | 0.569 | -0.007 (0.010) | 0.437 |
| E/e’ (n=2785) | 0.235 (0.079) ^**^ | 0.003 | 0.193 (0.071) ^**^ | 0.007 |
| Subclinical Atherosclerosis | | | | |
| CAC score (n=2616) | 21.494 (6.751) ^**^ | 0.001 | 27.841 (6.119) ^***^ | <0.001 |
| Renal Function | | | | |
| Log UACR (n=2794) | 0.079 (0.011) ^***^ | <0.001 | 0.076 (0.010) ^***^ | <0.001 |

Model 5 was adjusted for age, gender, race, smoking, drinking, BMI, educational attainment, SBP, LDL-c, HDL-c, average blood glucose, aspirin, medication for lower cholest, HTN and DM; Model 6 was adjusted for age, gender, race, smoking, drinking, BMI, educational attainment, SBP, LDL-c, HDL-c, Y25 blood glucose, number of measurements of FG, aspirin, medication for lower cholest, HTN and DM.

^***^, p <0.001; ^**^, p <0.01; ^*^, p <0.05.

**Supplemental Table 7 Logistic Regression Models to Examine the Associations Between Intensity of Glycemic Exposure During Young Adulthood with Target Organ Damage in Midlife**

| Outcome | Per 1 SD increment of IGE (standardized value) | | | | |
| --- | --- | --- | --- | --- | --- |
|  | N (%) | Model 1 | Model 2 | Model 3 | Model 4 |
|  |  | OR (95%CI) | OR (95%CI) | OR (95%CI) | OR (95%CI) |
| Cardiac Structure and Function  Structure impairment | | | | | |
| Left ventricular hypertrophy | 707 (24.7%) | 1.37 (1.25, 1.50) ^***^ | 1.18 (1.08, 1.29) ^***^ | 1.19 (1.05, 1.34) ^**^ | 1.25 (1.09, 1.43) ^**^ |
| Concentric remodeling | 393 (15.4%) | 1.05 (0.95, 1.15) | 0.99 (0.90, 1.10) | 0.98 (0.85, 1.12) | 0.93 (0.80, 1.09) |
| Systolic dysfunction | | | | |  |
| Abnormal ejection fraction | 23 (0.9%) | 1.31 (1.07, 1.60) ^**^ | 1.26 (1.02, 1.57) ^*^ | 1.10 (0.77, 1.57) ^*^ | 1.01 (0.69, 1.50) |
| Abnormal GLS> -11.10% | 121 (4.8%) | 1.35 (1.21, 1.52) ^***^ | 1.31 (1.16, 1.47) ^***^ | 1.22 (1.04, 1.43) ^*^ | 1.22 (1.02, 1.45) ^*^ |
| Diastolic dysfunction |  |  |  |  |  |
| Abnormal filling patterns | 347 (12.3%) | 1.09 (0.99, 1.20) | 1.08 (0.97, 1.20) | 0.98 (0.85, 1.14) | 0.92 (0.78, 1.08) |
| Increased filling pressure | 84 (3.0%) | 1.24 (1.09, 1.42) ^**^ | 1.18 (1.02, 1.36) ^*^ | 1.08 (0.91, 1.29) | 1.12 (0.92, 1.37) |
| Subclinical Atherosclerosis | | | | |  |
| Coronary artery calcium | 733 (28.0%) | 1.39 (1.27, 1.54) ^***^ | 1.34 (1.21, 1.47) ^***^ | 1.39 (1.23, 1.59) ^***^ | 1.24 (1.08, 1.43) ^**^ |
| Renal Function | | | | | |
| Albuminuria | 164 (5.9%) | 1.64 (1.49, 1.82) ^***^ | 1.60 (1.44, 1.77) ^***^ | 1.41 (1.23, 1.61) ^***^ | 1.40 (1.20, 1.63) ^***^ |

IGE, intensity of glycemic exposure.

All models were adjusted for covariates measured at Y25.

Left ventricular hypertrophy, Left ventricular mass >224 g for men and >162 g for women; Concentric remodeling, Relative wall thickness >0.42, Abnormal ejection fraction, ejection fraction<50%; Abnormal GLS, GLS>11.10%; Abnormal filling patterns, E/A≤0.8 OR ≥2, including impaired relaxation, pseudonormal, and restrictive filling; Increased filling pressure, septal E/e´ >15

Model 1 was adjusted for age, gender, race; Model 2 was additionally adjusted for smoking, drinking, BMI, educational attainment; Model 3 was additionally adjusted for SBP, LDL-c, HDL-c, Y25 blood glucose; Model 4 was additionally adjusted for aspirin, medication for lower cholest, HTN and DM.

^***^, p <0.001; ^**^, p <0.01; ^*^, p <0.05.

**Reference**

[1] C. Gatsonis, and A.R. Sampson, Multiple correlation: exact power and sample size calculations. Psychol. Bull. 106 (1989) 516-24.

[2] K. Krishnamoorthy, and Y. Xia, Sample Size Calculation for Estimating or Testing a Nonzero Squared Multiple Correlation Coefficient. Multivariate Behav Res 43 (2008) 382-410.

[3] D. Benton, K.J.C.S. Krishnamoorthy, and D. ANALYSIS, Computing discrete mixtures of continuous distributions: noncentral chisquare, noncentral t and the distribution of the square of the sample multiple correlation coefficient. 43 (2003) 249-267.

[4] J. Cohen, Statistical power analysis for the behavioral sciences, Academic press, 2013.
