# Supplementary figures and images for: Intensity of Glycemic Exposure in Early Adulthood and Target Organ Damage in Middle Age: The CARDIA Study
Source: Front Physiol. 2021 Jun 23;12:614532. doi: 10.3389/fphys.2021.614532 (PMC8260980; doi:10.3389/fphys.2021.614532)

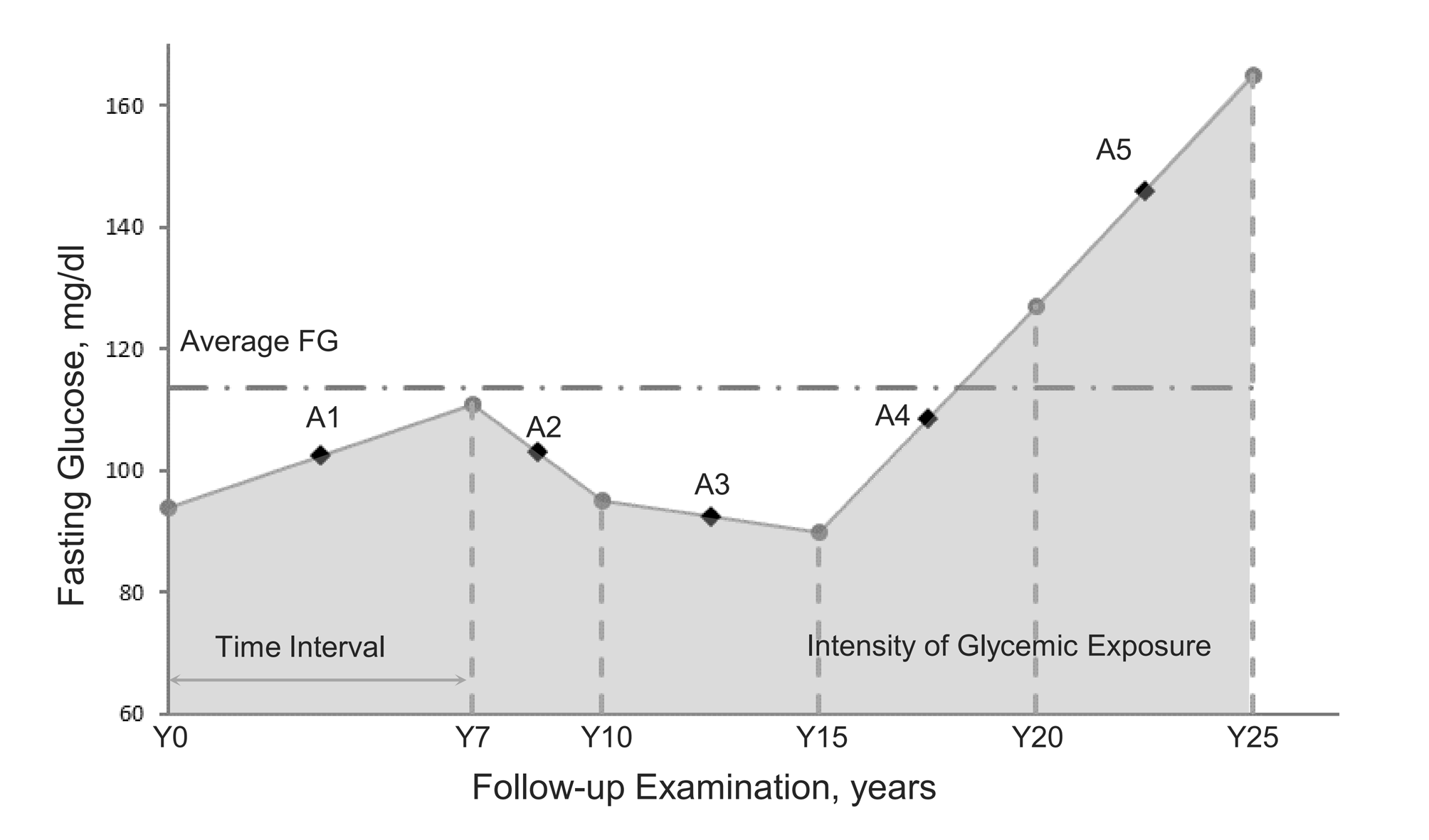

Supplement: Supplementary file 2 [file Image_1.TIF]
